# Elevated serum extracellular vesicle arginase 1 in type 2 diabetes mellitus: a cross-sectional study in middle-aged and elderly population

**DOI:** 10.1186/s12902-022-00982-z

**Published:** 2022-03-11

**Authors:** Xinwei Li, Wen Zhao, Lu Peng, Yu Li, Shaoping Nie, Huahui Yu, Yanwen Qin, Huina Zhang

**Affiliations:** 1 Beijing Anzhen Hospital, Capital Medical University, Beijing Institute of Heart Lung and Blood Vessel Disease, No. 2 Anzhen Road, Beijing, 100029 China; 2 Department of Emergency, Beijing Anzhen Hospital, Capital Medical University, Beijing, China

**Keywords:** Extracellular vesicles, Arginase 1, Type 2 diabetes mellitus

## Abstract

**Background:**

Serum extracellular vesicle (EV)-derived arginase 1 (ARG 1) plays a critical role in diabetes-associated endothelial dysfunction. This study was performed to determine the levels of serum EV-derived ARG 1 in T2DM and non-T2DM participants and to examine the association of serum EV-derived ARG 1 with T2DM incidence.

**Methods:**

We performed a cross-sectional study in 103 Chinese, including 73 T2DM patients and 30 non-T2DM. Serum EVs were prepared via ultracentrifugation. Serum EV-derived ARG 1 levels were measured by enzyme-linked immunosorbent assay. The correlations between serum EV-derived ARG 1 and clinical variables were analyzed. The association of serum EV-derived ARG 1 levels with T2DM was determined by multivariate logistic regression analysis. Interaction subgroup analysis was used to evaluate the interaction of the relevant baselines on the association between serum EV-derived ARG 1 levels and T2DM.

**Results:**

Serum EV-derived ARG 1 levels were significantly higher in T2DM patients compared with non-T2DM patients (*p* < 0.001). Correlation analysis revealed that serum EV-derived ARG 1 levels were positively associated with fasting plasma glucose (FPG) (*r* = 0.316, *p* = 0.001) and glycated hemoglobin (HbA1c) (*r* = 0.322, *p* = 0.001). Serum EV-derived ARG 1 levels were significantly associated with T2DM, especially in the subgroup of T2DM for more than 10 years (OR 1.651, 95% CI = 1.066–2.557; *P* value, 0.025), after adjusting for confounding factors.

**Conclusions:**

Elevated concentration of serum EV-derived ARG 1 is closely associated with T2DM.

**Supplementary Information:**

The online version contains supplementary material available at 10.1186/s12902-022-00982-z.

## Background

Type 2 diabetes mellitus (T2DM) has become one of the most pressing and prevalent epidemics in the last few decades and is associated with an increased risk of cardiovascular and cerebrovascular diseases with high mortality and disability [1, 2]. Therefore, the precise diagnosis of T2DM and T2DM-related diseases is particularly important. Currently, the use of body fluids, also called liquid biopsy, has attracted great interest for the quantification and specific biomarkers identification in circulating cells and circulating DNA and RNA, allowing a noninvasive or minimally invasive way to early diagnose tumors, to monitor real-time dynamics of cancer and patient follow up [3]. Meanwhile, clinical research has focused on the use of extracellular miRNAs or proteins as biomarkers as an alternative, noninvasive method for diagnosis and disease monitoring. Emerging evidence suggests that specific molecules derived from body fluids such as urine [4] and blood [5] are also closely associated with the presence of diabetes or diabetes-related diseases. As a new type of biomarker resource, extracellular vesicles (EVs) presented in accessible biofluids have garnered considerable attention from researchers due to their high stability in body fluids, their easy detection and their functional relevance in diseases [6, 7]. Growing evidence suggests that molecules in EVs are associated with diabetes or diabetes-related complications. For instance, miR-15a-5p and miR-15b-5p in urine EVs were found to be lower in diabetic individuals with renal disease [8]. In individuals with T2DM, platelet-derived EVs had higher expression of CD42 and CD41a, and monocyte-derived EVs had higher CD14 expression, compared with healthy controls [9]. Recently, a work suggested circulating miR-1281 as a potential biomarker of diabetic retinopathy [10].

ARG 1 is constitutively and most abundantly expressed in hepatocytes and functions as a detoxification enzyme in the urea cycle to remove ammonia [11]. Meanwhile, upregulation of ARG 1 contributes to vascular dysfunction by reducing l-arginine availability to nitric oxide (NO) synthase [12, 13]. Recently, red blood cell-secreted ARG 1 was demonstrated to induce endothelial dysfunction in T2DM individuals [14]. Interestingly, a chain of clinical evidence suggested that ARG inhibition improved endothelial function in the population of T2DM and T2DM combined with coronary artery disease [15, 16]. Our previous basic research indicated that serum EV- derived ARG 1 has a higher expression level in *db/db* mice and in diabetic patients compared to their counterparts [17]. The evidence led us to hypothesize that there should be a possible correlation between serum EV- derived ARG 1 and T2DM.

In the present study, serum EV- derived ARG 1 levels in T2DM patients and non-T2DM patients were detected by enzyme - linked immunosorbent assay (ELISA). And the association of serum EV- derived ARG 1 and T2DM was analyzed.

## Materials and methods

### Study design

This study was a cross-sectional study done in the Beijing Anzhen Hospital affiliated to Capital Medical University between 2018 and 2019. All participants gave written informed consent before enrollment, as appropriate. The protocol was approved by the Medicine Ethics Committee of Beijing An Zhen Hospital (No.2017005) and adhered to the Declaration of Helsinki.

### Study participants

251 participants were recruited from the endocrinology department and otolaryngological department of Beijing An Zhen Hospital. Non-diabetic patients were from clinical ENT (ear-nose-throat) inpatients and T2DM patients were from endocrinology inpatients. Patients with the following conditions were excluded: (1) Hepatic or renal dysfunction; (2) Diseases of the central nervous system, including stroke, Parkinson’s disease, multiple sclerosis, and traumatic brain injury; (3) Tumors, such as acute myeloid leukemia, neuroblastoma, squamous cell carcinoma, lung cancer and colorectal cancer; (4) Serious autoimmune and blood diseases; (5) Subjects with type 1 diabetes, gestational diabetes and immune system dysfunction were also excluded. Diabetes was defined if any of the following characteristics was observed [18]: history of physician-diagnosed diabetes, or use of medications or insulin for diabetes, or fasting plasma glucose (FPG) levels ≥7 mmoL/L (fasting time 8–12 h), or 2-h oral glucose tolerance test (OGTT) ≥ 11.1 mmoL/L, or random glucose levels ≥11.1 mmoL/L, or glycated hemoglobin (HbA1c) ≥ 6.5%. Prediabetes was defined as FPG levels from 5.6 to 6.9 mmol/L or OGTT levels from7.8 to11.0 mmol/L or HbA1c levels form5.7 to 6.4% [19]. Subjects whose fasting blood glucose < 6 mmol/L and all levels below the values above were enrolled in the non-T2DM group. In addition to meeting all diabetes indicators, newly diagnosed diabetes must also meet the requirement that they have not been treated with antihyperglycemic agents including insulin. Data on patient characteristics including clinical/ biochemical factors were collected. Demographic data, and laboratory parameters were obtained from clinical records. The remaining 103 patients (including 73 patients with T2DM and 30 patients without T2DM) between the age of 32 and 89 were included in the current study (Fig. S1).

### Anthropometric measuring

Anthropometric determinations and blood extraction were performed on a single day. Height and weight were measured with participants wearing light indoor clothing and barefoot using calibrated portable electronic weighing scales and portable inflexible height measuring bars. Blood pressure was measured after a 5-min rest in the sitting position and was determined at least three times at the right upper arm, and the mean value was used in the analysis. Body mass index (BMI) was calculated using the standard BMI formula: body mass (in kg) divided by the square of height (in m^2^). Nonsmokers were patients who had never smoked or had stopped smoking within ≥1 year before enrollment in the study. All remaining subjects were classified as smokers. Drinkers were defined as daily alcohol intake ≥ three times a week.

### Blood sample preparation and blood biochemical parameter measurement

The participants were asked not to eat after 21:00 the night before the session. An overnight fasting blood sample was drawn from each participant into a serum collection tube. Blood samples were then centrifuged for 10 min at 3000 g under 4 °C. Serum samples were subsequently stored in a freezer at − 80 °C within 2 h of collection. Samples were thawed at 4 °C prior to assay performance. Serum triglycerides (TG), total cholesterol (TC), low-density lipoprotein cholesterol (LDL-C), high-density lipoprotein cholesterol (HDL-C) and other serum biochemical indexes were measured by biochemical analyzer (Hitachi 7600, Tokyo, Japan) in the laboratory of Beijing Anzhen Hospital.

### EV isolation and identification

According to the previous literature with minor changes [20], serum EVs were centrifuged at 2000×g (10 min) and 12,000×g (45 min) to remove dead cells and cell debris after mixing 700 μL serum with equal-volume phosphate buffered saline (PBS). Supernatants were transferred to ultracentrifuge tubes, and EVs were precipitated at 110,000×g for 70 min (Beckman Coulter, CA, USA). The pellet, enriched with EVs, was resuspended in PBS and centrifuged again at 110,000×g for 70 min to obtain the purified EVs. All the above centrifugation steps were performed at 4 °C. The number and size of EVs was measured by Nanoparticle Tracking Analysis with a Nanosight NS300 instrument (Malvern, UK).

### Transmission electron microscopy

Serum-derived EVs were identified as previously described [17]. Microscopy images were captured by a transmission electron microscope (Hitachi H-7650, Tokyo, Japan) at an acceleration voltage of 80 kV.

### Measurement of serum EV-derived ARG 1

EVs extracted from 700 μL serum were melted in 200 μL PBS. Protease inhibitor cocktail (Thermo Fisher Scientific, Cat#: 87785) was added into the EV- containing PBS to protect proteins from degradation, followed by five times of freeze-thaw cycle (− 170 °C ∼ 37 °C) to rupture the membrane of EVs and release the inner protein. Serum EV-derived ARG 1 concentration was measured using Human Arginase 1 Simplestep ELISA Kit (ab230930, Abcam, Inc., Burlingame, CA), a highly sensitive enzyme-linked immunosorbent assay kit, according to the manufacturer’s instructions. Simply, 50 μL PBS with EV protein or standard was placed in each of the appropriate wells, and then 50 μL antibody cocktail was added to each well. Next, the plate was sealed and incubated for 1 h at room temperature on a flat shaker set at 400 RPM. Each well was washed three times with 1 × Washing Buffer. After the last wash, the plate was inverted and tapped gently with the clean tissue to remove excess liquid. Then 100 μL TMB Development Solution was added to each well and incubated for another 10 min in the dark on a flat shaker set at 400 RPM. The final step was to add 100 μL Stop Solution to each well and shake it on a flat shaker for 1 min to mix. The OD value at 450 nm was recorded by microplate reader (EnSpire, Perkin Elmer Singapore Pte. Ltd., Singapore).

### Western blotting

Protein samples prepared from serum or EV-free serum were separated by sodium dodecyl sulfate-polyacrylamide gels and transferred onto polyvinylidene fluoride membranes. The membranes were blocked with 5% fat-free dried milk in TBST at room temperature for 1 h and probed with the primary antibody ARG 1 (ab124917, Abcam, USA) overnight at 4 °C. Following incubation with specific horseradish peroxidase-conjugated secondary antibodies, chemiluminescence signals were visualized using supersensitive chemiluminescent substrates (Thermo Fisher Scientific, MA, USA) and detected by an imaging system (Bio-Rad, CA, USA).

### Statistical analysis

Statistical analyses were conducted using Statistical Package for the Social Sciences (SPSS) version 25.0 (IBM Corp; Armonk, NY). Continuous variables were expressed as mean ± standard deviation or median (interquartile range), and categorical variables were presented as numerals (percentage). Variables with a skewed distribution were log-normal transformed for these analyses. Baseline characteristics between the 2 groups were compared with the nonparametric Mann-Whitney U test, independent Student’s t-test, or the chi-square test. Linear correlations between EV-derived ARG 1 levels and other variables were assessed using Spearman’s rho correlation coefficients. The associations between serum EV-derived ARG 1 levels and T2DM were determined by multivariate logistic regression analysis. Differential expression of serum EV-derived ARG 1 between T2DM and non-T2DM was presented as a violin plot that was generated in R software, version 3.2.4. Interaction subgroup analysis was used to evaluate the interaction of the relevant baselines on the association between serum EV-derived ARG 1 levels and T2DM. *P* value < 0.05 was considered statistically significant.

## Results

**Fig. 1:**
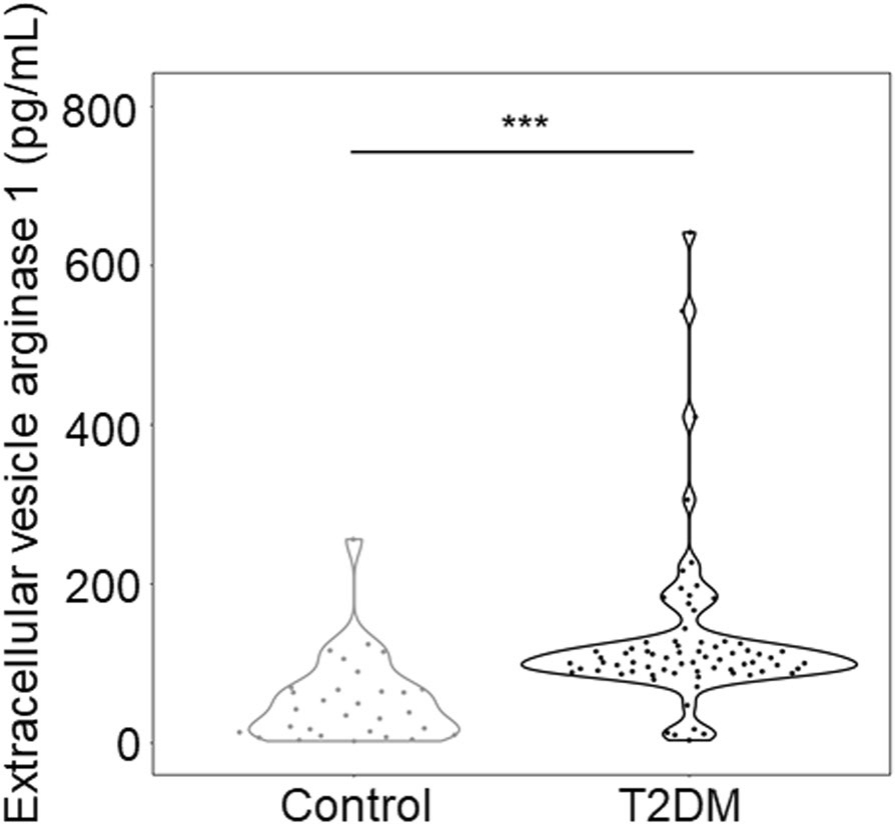
Serum EV-derived ARG 1 levels were higher in T2DM patients compared with non-T2DM patients. Serum EV-derived ARG 1 levels of T2DM group were higher than the non-T2DM group (104.99[90.05–126.10] vs. 40.50[12.67–68.05] pg/mL, *p* < 0.001). It showed the probability density of the data with different values on each side. *EV* Extracellular vesicles, *T2DM* Type 2 diabetes mellitus, *ARG 1* Arginase 1. **p < 0.001

EVs from non-T2DM and T2DM patients were isolated from serum by ultracentrifugation and visualized by transmission electron microscopy. Most of the serum EVs appeared intact with diameters less than 100 nm (Fig. S2 A and C). Nanoparticle tracking analysis showed that the average diameter of serum EVs was approximately 80 nm and that there was no significant difference in the size of serum EVs from patients with T2DM (Fig. S2D, 84.2 ± 37.1 nm) versus those from non-T2DM patients (Fig. S2B, 70.3 ± 7.9 nm). Whereas, the concentration of serum EVs was dramatically higher in T2DM (4.71*10^9^ particles/mL serum) compared to non-T2DM (4.38*10^8^ particles/mL serum) (Fig. S2E). Clinical and demographic features of the population in this study were summarized in Table 1. There were no differences in the parameters of sex, smoke, alcohol, high-sensitivity C-reactive protein (hs-CRP), triglycerides (TG), and homocysteine (Hcy) between T2DM and non-T2DM groups. Compared with the non-T2DM group, patients with T2DM had lower systolic blood pressure (SBP), diastolic blood pressure (DBP), HDL-C, LDL-C, higher age and a higher prevalence of coronary heart disease (72.86%). 38.36% T2DM patients were taking antihyperglycemic medication. As expected, the T2DM group had remarkably higher fasting plasma glucose (FPG) (*p* < 0.001), Glycated hemoglobin A1c (Hb1Ac) (*p* < 0.001). Of note, the levels of serum EV-derived ARG 1 were higher in the T2DM group compared with the non-T2DM group in this study (*p* < 0.001) (Fig. 1). Similarly, we found that ARG 1 protein levels were dramatically higher in the total serum of T2DM patients, but in EV-free serum it tended to increase in the T2DM group, despite the fact that ARG 1 expression was not statistically significant (Fig. S3–4).

**Table 1:** Baseline clinical characteristics of the study population

	T2DM (*n* = 73)	non-T2DM (*n* = 30)	*P* value
Age (years)	63.08 ± 9.25	56.00 ± 12.89	0.002*
Male (n, %)	45 (61.6%)	23 (76.7%)	0.144
Smoke (n, %)	27 (37.0%)	15 (50.0%)	0.222
Alcohol (n, %)	18 (24.7%)	10 (33.3%)	0.369
BMI (kg/m^2^)	26.00 ± 3.43	27.70 ± 4.71	0.042*
FPG (mmol/L)	8.60 ± 3.69	5.31 ± 0.43	< 0.001**
HbA1c (%)	7.50 (6.60–9.10)	5.75 (5.40–5.90)	< 0.001**
SBP (mmHg)	131.88 ± 15.47	142.33 ± 15.12	0.002*
DBP (mmHg)	74.38 ± 11.46	86.00 ± 15.41	< 0.001**
HDL-C (mmol/L)	0.97 (0.82–1.18)	1.15 (0.99–1.50)	0.004*
LDL-C (mmol/L)	2.20 ± 0.89	2.86 ± 0.83	0.001*
TC (mmol/L)	3.69 ± 1.06	4.66 ± 0.90	< 0.001**
TG (mmol/L)	1.45 (1.02–1.87)	1.42 (1.02–1.80)	0.616
Hcy (μmol/L)	11.80 (10.40–14.15)	11.10 (9.53–13.48)	0.372
hs-CRP (mg/L)	1.11 (0.59–3.33)	1.20 (0.45–5.25)	0.758
EV ARG 1 (pg/mL)	104.99 (90.05–126.10)	40.50 (12.67–68.05)	< 0.001**
Coronary heart disease (n, %)	51 (72.86%)	7 (22.33%)	< 0.001**
Diabetic medication before admission (n, %)	28 (38.36%)	0	

*EV* Extracellular vesicles, *T2DM* Type 2 diabetes mellitus, *ARG 1* Arginase 1, *SBP* Systolic blood pressure, *DBP* Diastolic blood pressure, *FPG* Fasting plasma glucose, *BMI* Body Mass Index, *HbA1c* Glycated hemoglobin A1c, *TC* Total cholesterol, *TG* Triglyceride, *LDL-C* Low-density lipoprotein cholesterol, *HDL-C* High-density lipoprotein cholesterol, *Hcy*, homocysteine, *hs-CRP* High-sensitivity C-reactive protein

Data are presented as n, mean ± SD, or median (interquartile range [IQR]) or n (%), unless otherwise stated. Differences between non-T2DM group and T2DM group were analyzed by the independent Student’s test, χ2 text, or Mann-Whitney U test

*p < 0.05, **p < 0.001

We investigated the correlation of serum EV-derived ARG 1 levels with clinical and biochemical variables using Spearman’s rho correlation analysis. Results showed that when all subjects were analyzed together, serum EV-derived ARG 1 levels were positively correlated with FPG (*r* = 0.316, *p* = 0.001) and HbA1c (*r* = 0.322, *p* = 0.001). In addition, serum EV-derived ARG 1 levels were negatively correlated with TC (*r* = − 0.242, *p* = 0.014). No correlations were found between EV-derived ARG 1 levels and age, SBP, LDL-C, HDL-C, DBP, BMI, TG, hs-CRP and Hcy, although negative correlation tendency was observed in SBP (*r* = − 0.185, *p* = 0.062) and LDL-C (*r* = − 0.186, *p* = 0.061). Furthermore, we calculated the correlations for the T2DM patients and non-T2DM patients, separately. The results showed that serum EV-derived ARG 1 were positively correlated with FPG (*r* = 0.388, *p* = 0.001) and HbA1c (*r* = 0.388, *p* = 0.001) levels in T2DM group, while the correlation was not observed in non-T2DM group (Table 2).

**Table 2:** Correlations of serum EV ARG 1 levels with other clinical variables

Parameters	Total	*P* value	T2DM	*P* value	non-T2DM	*P* value
Age (years)	−0.013	0.898	0.003	0.983	− 0.109	0.565
BMI (kg/m^2^)	0.010	0.924	0.075	0.527	−0.196	0.298
SBP (mmHg)	−0.185	0.062	−0.228	0.052	0.091	0.632
DBP (mmHg)	−0.088	0.375	−0.126	0.288	0.274	0.143
FPG (mmol/L)	0.316	0.001**	0.388	0.001**	−0.075	0.695
HbA1c (%)	0.322	0.001**	0.388	0.001**	−0.173	0.361
HDL-C (mmol/L)	−0.170	0.086	−0.173	0.143	−0.017	0.929
LDL-C (mmol/L)	−0.186	0.061	−0.229	0.052	0.113	0.553
TC (mmol/L)	−0.242	0.014*	−0.257	0.028	0.051	0.787
TG (mmol/L)	0.139	0.160	0.129	0.276	−0.072	0.705
Hs-CRP (mg/L)	0.052	0.599	0.113	0.343	−0.143	0.450
Hcy (μmol/L)	0.096	0.336	0.039	0.745	0.339	0.066

*EV* Extracellular vesicles, *T2DM* Type 2 diabetes mellitus, *ARG 1* Arginase 1, *SBP* Systolic blood pressure, *DBP* Diastolic blood pressure, *FPG* Fasting plasma glucose, *BMI* Body Mass Index, *HbA1c* Glycated hemoglobin A1c, *TC* Total cholesterol, *TG* Triglyceride, *LDL-C* Low-density lipoprotein cholesterol, *HDL-C* High-density lipoprotein cholesterol, *Hcy* homocysteine, *hs-CRP* High-sensitivity C-reactive protein

Spearman correlation test

Dependent variable: serum EV ARG 1

*p < 0.05, **p < 0.001

**Fig. 2:**
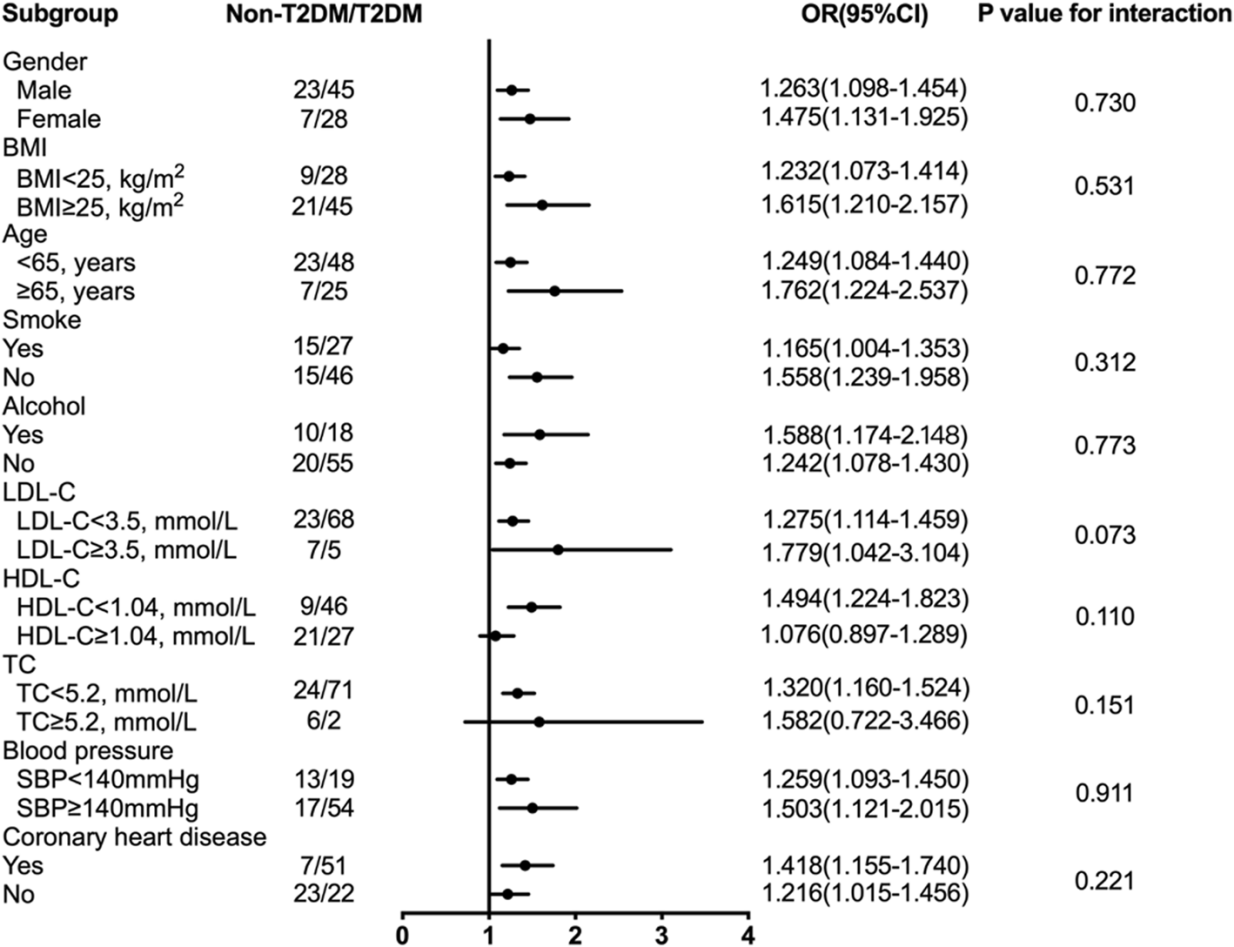
Subgroup analysis for interaction. *P* value is the value for interaction. *BMI* Body mass index, *LDL-C* Low-density lipoprotein cholesterol, *HDL-C* High-density lipoprotein cholesterol, *TC* Total cholesterol, *SBP* Systolic blood pressure *CAD* Coronary heart disease

We then constructed multivariable logistic regression models containing covariates to further evaluate the association of serum EV-derived ARG 1 levels and T2DM. Multivariate logistic regression analysis were performed in the total population as well as in subgroups classified by the duration of T2DM and medication. Results showed that serum EV-derived ARG 1 levels were significantly associated with T2DM, especially in the subgroup of T2DM for more than 10 years (OR 1.651, 95% CI = 1.066–2.557; *P* value, 0.025), after adjustment for age, sex, BMI, smoking and drinking history, SBP, LDL-C, HDL-C, TG, hs-CRP, and the comorbidities of coronary heart disease. However, no significant association between the levels of serum EV-derived ARG 1 and the prediabetic group was observed (Table 3). Meanwhile, we found that odds ratio was significantly higher in the drug treatment subgroup by 1.635 times (95% CI:1.139–2.347) compared to newly diagnosed T2DM, after adjustment for potential confounding factors (Table 4). To further investigate the possibility of relevant baselines attributed to the association between serum EV-derived ARG 1 levels and T2DM, we computed the odds ratios in the relevant subgroups and performed tests of interaction. Results presented that serum EV-derived ARG 1 was significantly associated with T2DM after considering all various covariates including gender, BMI, age, smoke, alcohol, LDL-C, HDL-C, TC, blood pressure and coronary heart disease. However, no specific various covariates could be observed having significant interaction (Fig. 2).

**Table 3:** Multivariate logistic regression analyses of serum EV ARG 1 levels and different duration of T2DM

EV ARG 1 (per 10 pg/mL increase)	No. of patients	Unadjusted		Model 1		Model 2		Model 3	
		OR (95%CI)	*P* value	OR (95%CI)	*P* value	OR (95%CI)	*P* value	OR (95%CI)	*P* value
Total	103	1.318 (1.165–1.492)	< 0.001	1.376 (1.194–1.585)	< 0.001	1.450 (1.135–1.851)	0.003	1.388 (1.155–1.669)	< 0.001
Non-T2DM (Reference)	18	1.000	–	1.000	–	1.000	–	1.000	–
Prediabetes	12	0.857 (0.711–1.034)	0.107	0.880 (0.729–1.063)	0.184	0.889 (0.694–1.139)	0.239	0.897 (0.696–1.157)	0.831
< 4, years	24	1.177 (1.007–1.374)	0.040	1.220 (1.025–1.453)	0.025	1.230 (1.014–1.492)	0.036	1.194 (1.001–1.424)	0.048
4–10, years	26	1.213 (1.051–1.400)	0.008	1.417 (1.065–1.886)	0.017	1.400 (1.071–1.829)	0.014	1.425 (1.098–1.850)	0.008
> 10, years	23	1.386 (1.101–1.743)	0.005	1.488 (1.109–1.997)	0.008	1.734 (1.040–2.890)	0.035	1.651 (1.066–2.557)	0.025

*EV* Extracellular vesicles, *T2DM* Type 2 diabetes mellitus, *ARG 1* Arginase 1, *SBP* Systolic blood pressure, *BMI* Body Mass Index, *TG* Triglyceride, *LDL-C* Low-density lipoprotein cholesterol, *HDL-C* High-density lipoprotein cholesterol, *hs-CRP* High-sensitivity C-reactive protein, *OR* Odds Ratio, *CI* Confidence Interval

Model 1: adjusted for age, sex, and BMI;

Model 2: adjusted for Model 1 + smoke, drink, SBP, TC, TG, hs-CRP

Model 3: adjusted for Model 2 + coronary heart disease

*p < 0.05, **p < 0.001

**Table 4:** Association of serum EV ARG 1 with diabetic medication and newly diagnosed T2DM in fully adjusted models

EV ARG 1 (per 10 pg/mL increase)	Newly diagnosed T2DM (*n* = 45)		T2DM with diabetic medication (*n* = 28)	
	OR (95%CI)	*P* value	OR (95%CI)	*P* value
Unadjusted	1.257 (1.110–1.422)	< 0.001**	1.366 (1.147–1.628)	< 0.001**
Model 1	1.290 (1.124–1.480)	< 0.001**	1.490 (1.201–1.849)	< 0.001**
Model 2	1.272 (1.086–1.490)	0.003*	1.563 (1.160–2.106)	0.003*
Model 3	1.335 (1.096–1.625)	0.004*	1.635 (1.139–2.347)	0.008*

*EV* Extracellular vesicles, *T2DM* Type 2 diabetes mellitus, *ARG 1* Arginase 1, *SBP* Systolic blood pressure, *BMI* Body Mass Index, *TG* Triglyceride, *TC* Total cholesterol, *hs-CRP* High-sensitivity C-reactive protein, *OR* Odds Ratio, *CI* Confidence Interval

Model 1: adjusted for age, sex, BMI;

Model 2: adjusted for Model 1 + age, sex, BMI, SBP, smoking, drinking, TC, hs-CRP and TG

Model 3: adjusted for Model 2 + coronary heart disease

*p < 0.05, **p < 0.001

## Discussion

In this cross-sectional study, we presented that serum EV-derived ARG 1 levels were significantly elevated in T2DM patients. Meanwhile, we revealed that serum EV-derived ARG 1 levels were independently associated with the presence and duration of T2DM after adjusting potential confounding factors.

Circulating EV contents in the body fluid reflect the changes in real time caused by a specific disease. For instance, endothelial EV-incorporated miRNAs such as miR-26a and miR-126 were significantly reduced in T2DM compared to non-diabetic patients [21]. Plasma EV-CD14 levels were found associated with a relative reduction for the development of T2DM after adjusting for traditional confounding factors in Second Manifestations of ARTerial disease study [22]. Here, we revealed that serum EV-derived ARG 1 was independently associated with the presence and duration of T2DM after adjustment for the confounding factors mentioned in this study.

ARG 1 not only acts as a key enzyme in the urea cycle in the liver, but also plays a pivotal role in the conversion of plasma l-arginine to urea and ornithine to decrease substrate availability for nitric oxide synthase, thereby affecting endothelial function [23, 24]. ARG activity was reported associated with diabetes or diabetes-related vascular complications. For example, a recent case-control study (including men and women from two different ethnicities) found that increased plasma ARG activity was correlated with the severity of hyperglycemia rather than plasma fasting insulin or free fatty acid levels in insulin-resistant type 2 diabetic subjects and age/weight-matched non-diabetic subjects [25]. Basic study of our team demonstrated that serum EV-derived ARG 1 has higher expression in *db/db* mice and in diabetic patients compared with their counterparts, which is involved in diabetes-induced endothelial dysfunction [17]. Likewise, in this study, the same elevated trends of serum EV-derived ARG 1 were evidently observed with the concentration almost 2.5-fold increase in diabetes patients. Meanwhile, the expression levels of EV-derived ARG 1 were positively correlated with FPG and Hb1Ac levels and independently associated with T2DM after adjusting for the confounding factors pointed out in this study.

Other studies proved that vascular ARG activity was also reported in hypertensive or apoE-deficient animal models [26, 27] or positively correlated with SBP in hypertensive animal models [28]. Whereas, our results demonstrated a negative correlation or tendency between the expression of serum EV-derived ARG 1 and TC or SBP in the general population and lower TC, LDL-C, and SBP levels in T2DM compared with non-T2DM subjects. The inconsistency existing between our findings and previous reports may be due to the clinical administration of cardiovascular drugs, such as statins [29], etanercept [30], diclofenac [31], cilostamide [32] and lisinopril [33] that influence ARG activity, but we did not successfully collect the information in this study.

We presented that serum EV-derived ARG 1 levels were significantly associated with T2DM by multivariate logistic regression analysis and noticed that the odds ratios gradually increased with the extension of T2DM course of time. Similarly, compared with the newly discovered T2DM group, we also found a higher odds ratio in the hypoglycemic drug treatment group. Considering the positive correlation between the duration of T2DM and diabetic medication, the consistent increase trends in the odds ratio of these two groups are reasonable. We observed a significant association between serum EV-derived ARG 1 levels and T2DM, no matter whether adjusting for the confounding factors or not. In line with this result, interaction subgroup analysis revealed that most of the relevant baselines including gender, BMI, age, smoke, alcohol, LDL-C, blood pressure, and coronary heart disease did not reverse the significantly associated trends between serum EV-derived ARG 1 levels and T2DM. Even in subgroups of female, BMI ≥ 25 kg/m^2^, ≥65 years old, drinker, LDL ≥ 3.5 mmol/L, SBP ≥ 140 mmHg and coronary heart disease, odds ratios were higher than their counterparts.

There are certain limitations in this study. Firstly, owing to the limited inclusion, non-diabetic patients in our recruitment had higher rates of smoking and drinking, a status that may be related to the higher blood pressure and hyperlipidemia in non-diabetic patients. Meanwhile, we did not investigate the effect of antihypertensive as well as lipid-lowering medications, partially reflected by the lower serum lipid profile and blood pressure in T2DM group on the levels of serum EV-derived ARG 1.The existence of all these unbalanced confounding factors might underestimate the difference in serum EV-derived ARG 1 levels between non-T2DM and T2DM patients, although we adjusted blood pressure and lipid levels and divided into subgroups in the latter multivariate logistic regression analysis to compensate these defects as much as possible. Secondly, we cannot exclude the possibility that the association of serum EV-derived ARG 1 with the presence of T2DM is related to other factors in the EVs and not only ARG 1 per se, which is worthy of further investigation in the future. Moreover, although a significant difference of serum EV-derived ARG 1 was obtained in T2DM and non-T2DM groups based on the limited sample size, large-scale studies are still required. In addition, we did not investigate whether the changes in serum EV-derived ARG 1 reflect the T2DM-associated vascular complications, it would be of great interest to the study. Finally, the important limitation of this study was its cross-sectional design, which makes it only be able to suggest associations rather than causal relationships between serum EV-derived ARG 1 and T2DM, and the limitation could be overcome by designing the study as a cohort (prospective or retrospective).

In conclusion, this study reports a significant association between serum EV-derived ARG 1 levels and the presence and duration of T2DM. It would help explain in part that more vascular diseases occur in T2DM compared to non-T2DM.

## Supplementary Information

**Additional file 1.**

## Data Availability

The data used and/or analyzed during the current study are available from the corresponding author on reasonable request.

## Abbreviations

EV: Extracellular vesicles
T2DM: Type 2 diabetes mellitus
ARG 1: Arginase 1
SBP: Systolic blood pressure
DBP: Diastolic blood pressure
FPG: Fasting plasma glucose
BMI: Body Mass Index
HbA1c: Glycated hemoglobin A1c
TC: Total cholesterol
TG: Triglyceride
HDL-C: HDL cholesterol
LDL-C: LDL cholesterol
Hcy: Homocysteine
hs-CRP: High-sensitivity C-reactive protein
OR: Odds Ratio
CI: Confidence Interval
