# Supplementary material for: Elevated serum extracellular vesicle arginase 1 in type 2 diabetes mellitus: a cross-sectional study in middle-aged and elderly population
Source: BMC Endocr Disord. 2022 Mar 11;22:62. doi: 10.1186/s12902-022-00982-z (PMC8917686; doi:10.1186/s12902-022-00982-z)
Supplement: Supplementary file 1 — Additional file 1. [file 12902_2022_982_MOESM1_ESM.docx]

Elevated serum extracellular vesicle arginase 1 in type 2 diabetes mellitus: a cross-sectional study in middle-aged and elderly population

Xinwei Li^1†^, Wen Zhao^1†^, Lu Peng^1^, Yu Li^1^, Shaoping Nie^2^, Huahui Yu^1^, Yanwen Qin^1^, Huina Zhang^1*^

^1^ Beijing Anzhen Hospital, Capital Medical University, Beijing Institute of Heart Lung and Blood Vessel Disease, Beijing, China

^2^Department of Emergency, Beijing Anzhen Hospital, Capital Medical University

Full list of author information is available at the end of the article

^†^Xinwei Li, ^†^Wen Zhao contributed equally to this work.

*Correspondence: Huina Zhang

Beijing Anzhen Hospital

Capital Medical University

Key Laboratory of Upper Airway Dysfunction-related Cardiovascular Diseases

Beijing Institute of Heart Lung and Blood Vessel Disease

No. 2 Anzhen Road, Beijing, 100029, China

Tel: +86-10-64456509

E-mail: [whinnerzhn@126.com](mailto:whinnerzhn@126.com)

**Supplementary Figure**

**Supplementary Figure 1** The flow chart of the study. 103 participants were consecutively enrolled including 30 non-T2DM patients and 73 T2DM patients. *EV* Extracellular vesicles, *T2DM* Type 2 diabetes mellitus, *ARG 1* Arginase 1, *HbA1c* Glycated hemoglobin A1c

**Supplementary Figure 2** The characterization of serum extracellular vesicles. **A and B** Transmission electron micrograph and the size of extracellular vesicles derived from non-T2DM serum. The scale bar represents 200 nm. **C and D** Transmission electron micrograph and the size of extracellular vesicles derived from T2DM serum. **E** Comparison of serum extracellular vesicle number in T2DM and non-T2DM patients. Results are the mean ± SD (n = 4). **p* < 0.05 vs. non-T2DM. *EV* Extracellular vesicles, *T2DM* Type 2 diabetes mellitus

**Supplementary Figure 3** ARG 1 protein expression levels in serum or EV-free serum from non-T2DM and T2DM patients. **A-D** ARG 1 protein levels in serum (**A and B)** or in EV-free serum (**C and D**) in non-T2DM and T2DM patients. The representative Western blots of three biological replicates were displayed. Results are the mean ± SD. **p* < 0.05 vs. non-T2DM. *EV* Extracellular vesicles, *T2DM* Type 2 diabetes mellitus, *ARG 1* Arginase 1

**Supplementary Figure 4** Replicates of Western blots for ARG 1 protein levels in serum or in EV-free serum. A (a1-a2, b1-b2, c1-c2) Replicates of Western blots for ARG1 protein levels in serum in non-T2DM and T2DM patients. B (a1-a2, b1-b2, c1-c2) Replicates of Western blots for ARG1 protein levels in EV-free serum in non-T2DM and T2DM patients. a1-a2, b1-b2, c1-c2 indicated three replicates with different exposure times. All the membranes were cut according to the molecular weight before incubation with primary antibody ARG 1 overnight at 4 °C.
